# Keeping the family in family planning

**DOI:** 10.1016/S2214-109X(14)70216-5

**Published:** 2014-06-25

**Authors:** Jenny Trinitapoli, Janneke Verheije, Susan Watkins, Sara Yeatman

**Affiliations:** Department of Sociology & Population Research Institute, Penn State University, PA, USA; Amsterdam Institute for Social Science Research, University of Amsterdam, The Netherlands; California Center for Population Research, University of California, Los Angeles, CA, USA; Department of Health and Behavioral Sciences, University of Colorado Denver, CO, USA

Maria Rodríguez and colleagues (March, p e131)^1^ posit that the term family planning is too antiquated and euphemistic to be useful and argue that it should be replaced with the more precise and scientific term contraception. We disagree that contraception is a better term and off er two simple insights to support this view.

First, the anthropological and demographic literature on fertility in Africa shows that the desire for a family factors heavily in young women's contraceptive choices.^2,3^ Our own work in Kenya and Malawi provides robust evidence that sexually active young adults use contraception to plan their families.^4,5^ Furthermore, many young people avoid modern contraceptive methods because they fear it will compromise their future fertility.^4^

Second, Rodríguez and colleagues' world in which all individuals make free and informed choices about contraceptive use is an imagined and profoundly western world, which ignores the social organisation of reproduction. That sex is a relational phenomenon (ie, by definition, at least one other person is included) is more than an inconvenient truth. Those lured by methodological individualism who continue to ignore men, relationships, and extended family networks do so at their own peril.^6,7^

We have no protective instinct towards family planning as a term or as a movement. But in our estimation, contraception is a far less accurate term to apply to what most young women across the globe are doing, even if the phrase is more palatable to western scientists.
